# Molecular Advances in Preeclampsia and HELLP Syndrome

**DOI:** 10.3390/ijms23073851

**Published:** 2022-03-31

**Authors:** Angeliki Gardikioti, Theodora-Maria Venou, Eleni Gavriilaki, Evangelia Vetsiou, Ioulia Mavrikou, Konstantinos Dinas, Angelos Daniilidis, Efthymia Vlachaki

**Affiliations:** 1 Hematological Laboratory, Second Department of Internal Medicine, Faculty of Health Sciences, School of Medicine, Aristotle University of Thessaloniki, Hippokration General Hospital, 54124 Thessaloniki, Greece; aggardik@auth.gr (A.G.); theoveno@auth.gr (T.-M.V.); evaggelia92@hotmail.com (E.V.); efivlachaki@yahoo.gr (E.V.); 2 Hematology & BMT Unit, General Hospital “George Papanicolaou”, 57010 Thessaloniki, Greece; molbiol.gpapanikolaou@n3.syzefxis.gov.gr; 3 Second Department of Obstetrics and Gyneacology, Faculty of Health Sciences, School of Medicine, Aristotle University of Thessaloniki, Hippokration General Hospital, 54124 Thessaloniki, Greece; dinas@auth.gr (K.D.); angedan@auth.gr (A.D.)

**Keywords:** ADAMTS-13, preeclampsia, complement system

## Abstract

Preeclampsia (PE) constitutes one of the principal reasons for maternal and perinatal morbidity and mortality worldwide. The circumstance typically implicates formerly healthful normotensive women, after 20 weeks of gestation, typically withinside the third trimester, without regarded threat elements or past deliveries. PE can be further complicated with hemolysis and thrombocytopenia, leading to the emergence of HELLP syndrome (Hemolysis, Elevated Liver enzymes, Low platelets). Both conditions are classified as hypertensive diseases of pregnancy (HDP), and their pathogenesis has been linked to an excessive maternal inflammatory response, accompanied by enhanced endothelial activation. Several studies have found that in pregnancies affected by PE/HELLP, von Willebrand factor (vWF) antigen levels (vWF:Ag) are significantly elevated, while its cleaving protease (ADAMTS-13, A Disintegrin-like and Metalloprotease with Thrombospondin type 1 motif, member 13) activity is normal to decreased. Furthermore, the higher urine excretion of the terminal complement complex C5b-9, as well as its greater deposition in the placental surface in preeclamptic women, imply that the utero-placental unit’s distinctive deficits are intimately tied to disproportionate complement activation. The goal of this updated evaluation is to provide the most up-to-date molecular advances in the pathophysiology of PE/HELLP syndromes. Recent medical data on vWF:Ag levels in patients with PE, ADAMTS-13, and dysregulation of the complement system, are highlighted and evaluated. Furthermore, we discuss the relationship between those entities and the progression of the disease, as well as their significance in the diagnostic process. Finally, considering the difficulties in analyzing and controlling those symptoms in pregnant women, we can provide a current diagnostic and therapeutic algorithm.

## 1. Introduction

Preeclampsia (PE) constitutes one of the major causes of maternal and perinatal morbidity and mortality worldwide. With its global incidence at 4.6%, which varies greatly among different countries and regions, it represents one of the four major reasons for maternal mortality, even in developed countries [1]. The condition usually implicates previously healthy normotensive women, after 20 weeks of gestation, most commonly in the third trimester, without known risk factors or past deliveries [2]. According to the International Society for the Study of Hypertension in Pregnancy (ISSHP), it manifests itself with hypertension and proteinuria and can progress to severe multisystem end-organ damage, resulting in liver or renal failure, convulsive activity (eclampsia), acute respiratory distress syndrome (ARDS) due to pulmonary edema or intrauterine growth restriction (IGR). PE can be further complicated with hemolysis and thrombocytopenia, leading to the emergence of HELLP syndrome (hemolysis, elevated liver enzymes, low platelets), with distinctive right upper quadrant abdominal pain [3]. Both entities are included in the scope of hypertensive disorders of pregnancy (HDP), but their exact etiology remains elusive, although multiple studies suggest their multifactorial (maternal and fetal, genetic, and environmental) background [4]. Chronic hypertension, gestational hypertension, and finally, chronic hypertension with superimposed preeclampsia are the other three clinical entities, constituting the general category of hypertensive disorders of pregnancy [5].

The pathophysiology of PE is complex, with the precise underlying cause not fully elucidated, although probably heterogeneous. During PE, the syncytiotrophoblast is damaged, which has been suggested to lead to a compensatory increase in cytotrophoblast proliferation, resulting in poor differentiation of cytotrophoblast cells [6]. PE is also associated with maternal and placental vascular dysfunction, from placentation to beyond delivery. These defects have been attributed to various factors, including endothelial dysfunction, insufficient trophoblast invasion, oxidative stress, and poor placental oxygen extraction. It is uncertain whether the development of vascular impairments in PE is a result of underlying vascular pathology or exclusively a result of elevated trophoblast stress signals to the mother [7]. The PE and/or HELLP syndrome is correlated with, or attributed to, an exaggerated maternal inflammatory process, with increased endothelial activation. Numerous studies report that von Willebrand factor (vWF) antigen levels (vWF:Ag) are significantly increased, while its cleaving protease (ADAMTS-13, A Disintegrin-like and Metalloprotease with Thrombospondin type 1 motif, member 13) activity is normal to decreased in pregnancies affected by PE/HELLP [8,9]. Von Willebrand factor (vWF) is a cardinal mediator for the adhesion and aggregation of platelets’ surface to the subendothelial matrix under shear stress. The vWF is synthesized from megakaryocytes and endothelial cells, in a primal form of large multimers that undergo proteolysis into smaller derivatives, under the cleaving assistance of ADAMTS-13. As it is known from the pathogenesis of thrombotic thrombocytopenic purpura (TTP), the inhibited expression of ADAMTS-13 leads to the impairment of vWF cleavage, which, in turn, results in the development of disseminated platelet-rich thrombi in the circulation and the consequent activation of the inflammatory course [10]. Because thrombotic microangiopathies (TMAs) and PE/HELLP syndrome present with similar clinical manifestations in pregnancy, such as end-organ failure due to ischemia, the irregularities in the proteolysis of vWF and the reduced or false expression of ADAMTS-13 are examined thoroughly, regarding their involvement in the development of PE and HELLP syndrome [8,11].

Another key aspect of the pathogenetic mechanisms entailed in the development of PE and HELLP syndrome is the role of the complement system [12]. The complement system is activated and strictly regulated through the classic, lectin, or alternative pathway, as an integral part of the innate immune response to a number of pathogens or damaged cells. Normally, there is some degree of complement activation in pregnancy, which, however, is kept under control, as the placenta expresses complement-inhibiting proteins to sustain the maternal immunological tolerance [4]. Research has suggested that the characteristic of insufficient placental perfusion and the consequent impairments in the utero-placental unit are inextricably linked to disproportionate complement activation, as proven by the elevated urine excretion of the terminal complement complex C5b-9, as well as its increased deposition in the placental surface in preeclamptic women [3]. A great scientific effort is currently ongoing and contributes to the clarification of what triggers the imbalanced complement cascade, whether it is a genetic deficiency in the regulation of the complement system or a maternal immune reaction to the placental cells, or most likely, a combination of both [4].

The purpose of this state-of-the-art review is to present the most recent molecular advances in the pathogenesis of PE/HELLP syndromes, recent scientific data regarding the increased von Willebrand factor (vWF) antigen levels (vWF:Ag) in patients with PE, ADAMTS-13, and the dysregulation of the complement system will be highlighted and analyzed. Furthermore, we will discuss the correlation between these entities in the development of the disease, as well as their significance in the diagnostical process. Finally, taking into account the difficulties in diagnosis and management of these syndromes in pregnant women, we will suggest an updated diagnostic and therapeutic algorithm.

## 2. Results

### 2.1. ADAMTS13 and vWF in Preeclampsia/HELLP Syndrome

Von Willebrand(vWF) is a multimeric protein, of high molecular weight, whichcontributes to normal hemostasis by recruiting platelets from rapid blood flow sites to vessel injury sites. vWF is usually proteolytically cleaved and regulated by ADAMTS13 metalloprotease. In the presence of anti-ADAMTS13 autoantibodies or low ADAMTS13 activity, there is a concomitant accumulation of high-molecular-weight vWF multimers, increasing platelet aggregation, eventually leading to the occlusion of small vessels [11].

Several studies investigated the relationship between the levels of ADAMTS13 activity and the development of PE/HELLP syndrome. It was interesting to find several studies with a statistically significant decline in ADAMTS13 activity in women with PE, relative to healthy controls [2,13,14,15,16,17,18]. Accordingly, Sabau et al. measured serum ADAMTS13 activity in a group of pregnant women with HELLP syndrome and found that it was low (<30%) in 13% of the sample [16]. The expression of placental ADAMTS13 has also been evaluated in PE and hypoxia, with a significant reduction in expression, in contrast with other metalloproteinases of the same group (ADAMTS1, 4 or 12) [14]. Overall, placental ADAMTS13 expression is reduced during pregnancy, especially during the first gestational semester, with a statistically significant difference among preeclamptic and normotensive pregnant women [18]. ADAMTS13 low levels were explicitly associated with early-onset PE and were associated with the level of inflammation [15].

In contrast, only a few papers reported no differences between the two groups, suggesting that ADAMTS13 activity is not significantly associated with PE [19,20,21]. In the study of Yoshida et al., pregnant women with HELLP syndrome were also included in the sample, but the sizes of the patient group (*n* = 13) and the control group (*n* = 128) are obviously inequivalent [20] Interestingly, according to von Krogh et al., no statistically significant differences were detected concerning lower levels, genotype, allele, and haplotype of the ADAMTS13 variants between the preeclamptic and the healthy control group of pregnant women [21].

In the literature, conflicting findings have been reported regarding vWF levels, leading to inconclusive results. The study of Stepanian et al. featured a larger sample, investigating a distinguished number of 140 preeclamptic pregnant women and compared them with an arithmetically equal control group and did not detect any statistically significant difference between both groups, concerning vWF [14]. On the contrary, numerous other studies have demonstrated a statistically significant increase in the group of pregnant women with PE or HELLP syndrome [2,15,18,19,20,22,23]. In all of them, the measurement of vWF was realized using an enzyme-like immunosorbent assay (ELISA), except for one study, in which an immunoturbidimetric assay was performed for that purpose [2]. It should be noted that the discrepancy in the sample size among different studies complicates the comparison and the extraction of reliable conclusions. In the study conducted by Molvarec et al., preeclamptic women had a higher concentration of vWF: Ag but a normal level of multimers compared to a healthy control group [22]. Chen et al. stated that the increased levels of vWF persist during the first week after delivery in preeclamptic women [24].

In the study of Hulstein et al., there was a statistically significant increase in vWF in the group of women with HELLP syndrome compared with the preeclamptic group. Conversely, no statistically significant difference was found between both groups, regarding ADAMTS13 levels [18].

It is also important to identify fluctuations in ADAMTS13 and vWF levels or modification of their function during normal pregnancy. According to Chen et al., in normal pregnancy, vWF is characterized by an enhanced adhesion ability, resulting in a pregnancy-related hypercoagulable state [24]. Moreover, in the study of Drury Stewart et al., a statistically significant increase in vWF: Ag (vWF antigen) was described in normal pregnancy, while vWFpp (vWF propeptide) also increased at a later stage of gestation [23]. In the same study, no statistically significant elevation of ADAMTS13 was detected. Accordingly, Alpoim et al. found a statistically significant difference between normotensive pregnant women, in comparison with a group of non-pregnant women. Furthermore, there was no high-molecular-weight or structurally and functionally defective vWF multimers [15]. Moreover, according to Yoshida et al., vWF: Ag levels started to increase after the first semester, nearly doubled at the beginning of the last gestational semester, and suddenly decreased right after delivery, in a study group of healthy pregnant women. In the same study, PE and HELLP syndrome were correlated with increased values of VWF: Ag with a statistically significant difference between HELLP syndrome and normal pregnancy (*p* < 0.05) [20].

In the study of Molvarec et al., no statistically significant difference was found in ADAMTS13 activity, when the preeclamptic group was compared with a group of non-pregnant women. In the same study, it was stated that significantly higher values of ADAMTS13 were detected in multiparas, in comparison with primiparas, in all three investigated groups (preeclamptic, healthy pregnant, non-pregnant) [19]. On the other hand, there are a couple of studies, according to which there was a significant decrease in ADAMTS13 and an increase in vWF levels between preeclamptic and non-pregnant women [2,15]. All bibliographic data are concentrated and presented in Table 1.

### 2.2. Complement in Preeclampsia/HELLP Syndrome

As an initial defensive mechanism against various antigens through opsonizing proteins, inflammation, and cell membrane assault, the complement cascade is an essential component of the human innate immune system. Complement activation is triggered by enzymatic cleavage via the classical, the lectin, and the alternative pathway. C3 is a common converging protein of the three pathways, which leads to the formation of components C3a and C3b. Deposition of C3b by the classic or lectin pathway also facilitates the amplification of the alternative pathway, enhancing C3b deposition through a positive loop. As a result, the constant deposition of C3b generates the C5 convertase, which signals its cleavage to elements C5a and C5b (anaphylatoxins). C5b then binds to C6, C7, and multiple molecules of C9, forming the terminal complement complex C5b-9, known as the “membrane attack complex” (MAC) [25].

**Figure 1:**
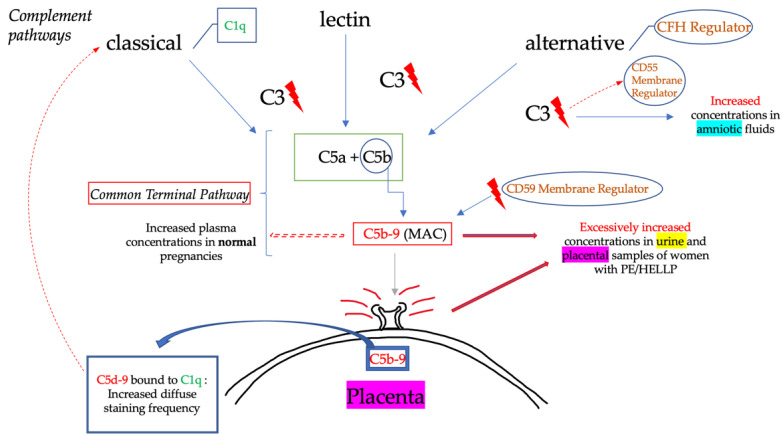
Complement cascade dysregulation in preeclampsia and HELLP syndrome. (CFH, Complement Factor H; C1q, C3, Complement proteins; CD55, CD59, Complement Regulators; C5a, C5b, Complement proteins /Anaphylatoxins; MAC, Membrane Attack Complex).

Preeclampsia (PE) is a complicated cardiovascular condition of pregnancy, with diverse pathogenic mechanisms at its root. While increased levels of the anti-angiogenic factors, such as sFlt1 (soluble fms-like tyrosine kinase 1), and decreased levels of angiogenic factors, such as placenta growth factor (PlGF) and vascular endothelial growth factor (VEGF), are associated with hypertension and proteinuria and, subsequently, the development of PE [26], increasing evidence suggests that the terminal complement proteins C5a and C5b-9 are also involved in this process. The complement system seems to be fundamental in the inflammatory process of PE and HELLP syndrome, with various studies demonstrating that PE is eventually a complement-mediated disease (Figure 1). Elevated amounts of complement components and their activation derivatives, including C5a, and C5b-9 complex, have been detected in the circulation of PE patients compared to uncomplicated pregnancies, albeit the data in this field are highly contradictory [25]. Complement activation products were also discovered in the urine of severe PE patients and are thought to be a sign of complement-mediated kidney injury [27].

In 2008, Rampersad et al. detected increased trophoblast deposition of the terminal complement component C5b-9 in placental sections of preeclamptic women. The study seemed to enhance apoptotic cell death, which increased up to 2-fold in the preeclamptic samples compared to the healthy ones [28]. Additionally, Banadakoppa et al., in 2015, observed significant amniotic C3 levels in women who developed early-onset PE before 34 gestational weeks [29]. The involvement of the complement system in the hemolysis of severe PE and HELLP syndrome was also showcased in the study of Vaught et al., in which the complement activity was identified through a modified Ham test in ex vivo experiments [30]. Important evidence was exhibited in the research of Burwick et al., which showed a 5-fold rise in C5a levels in women with severe PE, compared to matched healthy controls in urine investigations. Urine C5b-9 levels were also found to be more than 4-fold higher in women with severe PE, according to three distinct studies. In contrast to plasma C5b-9 levels (which are also found to be increased in the plasma of women with normal pregnancy at 36 to 37 weeks gestation), women with severe PE had significantly higher urine C5b-9 levels [31]. In a multicenter observational study, urinary excretion of C5b-9 was found in 96% of cases of severe PE, 12% of chronic hypertension cases, and 8% of healthy controls, leading to the distinguishment of severe PE from other hypertensive diseases of pregnancy [32].

Whereas C5b-9 trophoblast deposition in preeclamptic placentas appears to be higher, it is unclear whether this is due to excessive complement activation or defective control. Buurma et al. discovered that placental complement-regulating factors CD55 and CD59 were elevated 2-fold and 4-fold, respectively, in the placentas of women who delivered with preeclampsia compared to uncomplicated births. Researchers reasoned that the upregulation of CD55 and CD59, in response to enhanced complement activation, indicates the presence of a positive loop to preserve trophoblast viability [33].

Although C5b-9 is the common terminal effector of all complement pathways, the aim has been to investigate if placental C5b-9 deposition is triggered by a particular activation mechanism. Buurma et al. also detected a higher staining frequency of placental C4d in preeclamptic placentas [34]. As C4d is a shared component of both the classical and the lectin activation pathway, the researchers used diffuse staining at the syncytiotrophoblast surface. Therefore, it was observed that C4d bound to component C1q, suggesting that C4d concentrations were most probably a product of classical pathway activation [33]. However, in 2020, He et al. indicated impairments of the alternative pathway, as a deregulated mechanism of complement activation, involved in PE and/or HELLP syndrome. The study recognized upregulation of the complement factors H and B (CFH, CFB) of the alternative pathway, in plasma samples of preeclamptic patients, specifically with early-onset PE (at 6 to 8 gestational weeks) [34]. These findings may suggest the different underlying pathogenetic processes behind early- and late-onset PE; however, more research is required to validate their clinical significance.

Over the last decade, a lot has been discovered about complement gene mutations, due largely to a better knowledge of atypical hemolytic uremic syndrome (aHUS), which is also a complement-mediated condition, and its clinical phenotype usually overlaps with HELLP syndrome, indicating a shared biological basis [30]. Specifically, in a study by Vaught et al., significant similarities of variants in genes that control the alternative pathway activation of the complement system were discovered between aHUS and HELLP syndrome, using phenotypic and functional mHam tests and setting strict criteria for the included variants. Using this tight criterion, the HELLP syndrome cohort had considerably more alternative pathway germline mutations than controls (46% vs. 8%, *p* = 0.01), whereas there was no difference when compared to aHUS [30]. These findings support earlier research that suggests complement system plays a key role in the pathogenesis of PE/HELLP syndrome. For example, in 2007, Fang et al. had evaluated the gene variant A304V of the complement-modulating membrane cofactor protein (MCP), known to be associated with aHUS, in a patient with HELLP syndrome [35]. The variant was found to reduce the regulating functionality of the MCP, regarding the complement activation in situ. Subsequent studies of MCP mutations in PE and HELLP syndrome, although with ranging prevalence, also detected defective variants in these conditions [34]. Furthermore, Vaught et al. described rare variants and deletions of CFH-related (CFHR) genes. CFHR gene paralogs were identified in 21% of partial HELLP cases and 45% of HELLP cases, leading to the conclusion that genetic variants predispose some women to manifest PE and/or HELLP syndrome [36].

Overall, in many women who develop preeclampsia and HELLP syndrome, complement control is disrupted, and terminal complement activation is enhanced, according to the available research. These data imply that inhibiting the terminal complement pathway (e.g., C5 blockage) may be a potential disease-mitigation approach. In this regard, a C5-activation inhibitor (Eculizumab) has been utilized in a single case report by Burwick et al., to off-label treat PE/HELLP at 27 weeks gestation, achieving 17 days of prolongation of pregnancy and minimizing neonatal morbidity [37], while it has been more extensively used as a therapeutic option in aHUS—complicated pregnancies [25]. This idea has been experimentally implemented in a currently phase 1 clinical trial (NCT Number: NCT04103489), in which investigators propose the therapeutic use of Eculizumab for women with HELLP syndrome at 23–30 weeks gestation. The researchers indicate that their study was based on the finding that around 50% of women with HELLP syndrome carry alternative pathway-related mutations, that affect regulating proteins, and which are remarkably similar to those associated with aHUS [38].

To date, research reveals that the degree of complement activation is mainly comparable in preeclampsia with severe symptoms and HELLP syndrome, which is compatible with the existing knowledge of the disease spectrum. There is no evidence that HELLP syndrome is separate from preeclampsia; instead, the two disorders share pathophysiologic features, with excessive complement activation being a common hallmark [38,39]. The involvement of complement proteins in preeclampsia and HELLP syndrome is now apparent, but additional research is needed to understand how the complement protein biomarkers and complement genetic variations might be used therapeutically, to assist illness prediction, diagnosis, and therapy [39].

### 2.3. Complement and von Willebrand Factor Interaction

The formation of C5b-9 on human endothelial cells leads to the increased secretion of high molecular weight vWF multimers and the generation of membrane particles from the endothelial cell surface, which express binding sites for activated factor V (Fva), promoting prothrombinase activity [28]. Furthermore, ultra-large vWF provide an activating surface for the assembly of the alternate pathway convertase, complement factor H (CFH); FH may decrease ultra-large vWF to smaller forms, although the interaction remains elusive. Consequently, a defective function of CFH may also be linked to the development of PE through a defective regulation of vWF cleavage [25].

## 3. Discussion

### 3.1. Differential Diagnosis of TMAs

Thrombotic microangiopathies (TMAs) constitute a group of clinical entities with a similar pattern of clinical manifestations and, more specifically, the combination of different organ dysfunction, low platelet counts, and microangiopathic hemolytic anemia. HELLP syndrome and TTP have been correlated with different pathogenetic mechanisms, belonging, however, to the general category of TMAs [39].

Apart from those clinical entities, there are other primary and secondary pathological conditions falling under the general category of TMAs, such as drug-induced and transplantation or malignancy-related TMAs, Uremic Hemolytic Syndrome, conditions related to autoimmune diseases (scleroderma, Systemic Lupus Erythematosus), malignant hypertension and, finally, Disseminated Intravascular Coagulation [39].

The initial step of the diagnostic process is the exclusion of clinical entities, according to the medical record of the investigated patient, for example, the absence of drug intake, transplantation history, or autoimmune diseases [40]. However, the differential diagnosis among different conditions, belonging to the broad spectrum of TMAs, constitutes a challenge in everyday clinical practice and cannot always be based exclusively on clinical manifestations. Therefore, during the past decades, there has been much interest in understanding the pathogenetic mechanisms of TMAs and detecting biomarkers that can lead to a safe and definite diagnosis and prognosis of the clinical course [39].

More specifically, the initial steps of the diagnostic process should be orientated in clinical conditions commonly seen in pregnancy, such as acute fatty liver of pregnancy, preeclampsia, preeclamptic toxemia, eclampsia, and HELLP syndrome. Other clinical entities, which should also be excluded, are antiphospholipid syndrome, systemic lupus erythematosus, disseminated intravascular coagulation, and sepsis [40].

First, the acute fatty liver of pregnancy is characterized by an elevation in conjugated bilirubin, along with intense pain, situated on the upper right abdominal side and is usually a diagnosis based on clinical criteria [40,41]. Weight loss, increased urination, polydipsia, decreased glycose serum levels, along with prolongation in prothrombin time, low factor V and antithrombin levels, and signs of kidney dysfunction, coexist in most of the cases [40]. Echo-Doppler is useful to exclude the possibility of Budd–Chiari syndrome [41].

Moreover, specific laboratory and clinical criteria exist for the diagnosis of antiphospholipid syndrome and systemic lupus erythematosus (International consensus Sydney classification criteria, 2019 EULAR/ACR classification criteria). Sepsis with multiple organ dysfunction should be suspected in the presence of relevant conditions (chorioamnionitis, endometritis, urinary tract infection, severe inflammatory syndrome) and is diagnosed with the conduction of blood cultures, even if high fever is absent [41].

Uremic hemolytic syndrome is frequent in the pediatric population and shares some basic laboratory and clinical characteristics with TTP (jaundice with free bilirubin elevation, liver enzymes and LDH elevation, presence of schistocytes in peripheral blood, extreme thrombocytopenia). The main difference between those two clinical conditions is the predominance of neurological symptoms (blurry vision, convulsions, paresis) in TTP, in contrast with the profound kidney dysfunction in patients with TTP [40]

Furthermore, HELLP syndrome is characterized by an increase in body weight, edema, hypertension, profound urine protein, liver enzymes, and LDH elevation. In Table 2, we present the Mississippi triple-class and Tennessee systems used to classify patients with HELLP syndrome [42]. More interestingly, it appears that the key to the differential diagnosis between TTP and HELLP syndrome is ADAMTS13 activity, which is a biomarker widely used in clinical practice [41].

### 3.2. Role of ADAMTS13 and vWF in the Differential Diagnosis

Differential diagnosis between TTP and HELLP syndrome/preeclampsia is crucial because of the different management options. More specifically, delivery is considered the treatment of choice for managing HELLP syndrome and preeclampsia, and a disease resolution is expected 1–2 days postpartum. Complement inhibition may also be beneficial in the broad spectrum of complement-mediated TMAs, including HELLP syndrome. Nevertheless, delivery is not thought to be an acceptable treatment for TTP, as symptoms keep deteriorating postpartum, unless plasmapheresis is performed [41].

In the literature, there are many published case reports, in which TTP was diagnosed postpartum due to the persistence of the disease. Moreover, HELLP syndrome or preeclampsia should also be considered in pregnant women with a personal history of hereditary or acquired TTP. It should also be noted that the coexistence of TTP and PE/HELLP syndrome should not be excluded from the beginning [42,43,44,45,46]. Otherwise, PE/HELLP are thought to be more frequent in patients who have already recovered from TTP, or even induce the development of TTP in predisposed pregnant women [42,45,46]. Although delivery is not mandatory in TTP, it is required when TTP is combined with preeclampsia, even at a gestational age before 34 weeks [47].

The first steps of the diagnostic process should be immediate, based on the co-estimation of personal history, clinical manifestations, and basic laboratory results, since primary intervention plays a critical role in patients’ survival. Early gestational age (<20 weeks) can arouse suspicion that the issue is TTP. Nevertheless, it is sometimes difficult to discriminate TTP from HELLP during the third trimester of pregnancy, as there is an overlap in clinical manifestations and laboratory parameters [42]. Hemolysis, low platelets count, hypertension, renal dysfunction, increased liver enzymes and LDH can be found in both clinical entities. On the other hand, the coexistence of seizures, mobility issues, reddish urine, prominent anemia with the presence of schistocytes, and decreased haptoglobins with mild liver enzymes elevation (LDH/AST > 22.12), severely low level of platelets, and fever is more indicative of TTP [41].

ADAMTS13 activity is a key diagnostic tool, since ADAMTS13 deficiency (<20%) confirms TTP diagnosis. In the literature, the cut-off value, below which the diagnosis of TTP can be considered definitive, varies in range, between 5–20% (10% is the most frequently reported) [40,41,42]. In the review of Pourrat et al., it is stated that the measurement of ADAMTS13 activity is considered a reliable practice for the differential diagnosis between TTP and HELLP syndrome. ADAMTS13 is undetectable in TTP, whereas lower levels are found in patients with HELLP syndrome. ADAMTS13 activity has been found to be 31% (12–43%), whereas the expected average value of ADAMTS13 in late pregnancy is higher (71%) [41]. In Table 3, HELLP syndrome and TTP are compared, concerning different clinical and laboratory parameters [41].

The question is whether ADAMTS13 could be considered a reliable biomarker for the diagnosis of TTP, since there are several studies supporting a statistically significant decrease in ADAMTS13 in other TMAs, such as HELLP syndrome/preeclampsia [2,17,18,19,20,21,22,24]. ADAMTS13 activity values can vary from 12% to 43% in HELLP syndrome. Consequently, even if a deficient level of ADAMTS13 activity is highly diagnostic of TTP, a more average one cannot exclude the possibility of HELLP syndrome/preeclampsia. Nevertheless, it is believed that a complete absence of ADAMTS13 activity is not compatible with the diagnosis of HELLP syndrome/preeclampsia [46]. The differential diagnosis in patients with average levels of ADAMTS13 activity is usually based on clinical parameters, and further research is needed to establish the use of new and more reliable biomarkers.

On the other hand, whilst the complement system is a crucial part of the inflammatory process of PE and increased amounts of complement components and their activation products, such as C5a, and C5b-9 complex, have been documented in the circulation of PE patients when compared to normal pregnancy, results remain diverse and larger samples are required [33]. Complement activation products have also been discovered in the urine of severe PE, and they are thought to be a sign of complement-mediated kidney injury [27,29]. Moreover, increased levels of the alternate route activation fragment Bb have been proposed to predict PE development [27]. These suggestions, along with the genetic findings that indicate the existence of mutations that facilitate the excessive or unregulated activation of the complement’s alternative pathway [38], might constitute, in the future, solid predictive and prognostic factors for an earlier diagnosis of PE and HELLP syndrome.

In addition, in everyday clinical practice, there are some cases where the measurement of ADAMTS13 is not possible or immediate action is needed. Therefore, initial diagnosis and, by extension, the choice of the most appropriate treatment among the available options, is based exclusively on clinical and basic laboratory parameters. Table 4 presents data from several case reports, collected from our literature research process, indicating the difficulties in setting the definite diagnosis. It should be noted that there are many cases in which the final diagnosis remains vague, or the authors attribute the unsuccessful initial management to a possible coexistence of HELLP/PE and TTP from the beginning [43,44,45,46,47,48].

### 3.3. Proposed Algorithm for the Differential Diagnosis and Management of Pregnant Women with TMA

#### 3.3.1. Diagnosis

First, detailed medical history plays a crucial role in the differential diagnosis between different clinical entities. Personal history of preeclampsia, thrombophilia, or chronic hypertension, primiparity, very young age or age over 35 years old, obesity and multiple pregnancy is thought to predispose one to the development of preeclampsia. In fact, preeclampsia does not only occur in previously healthy pregnant women, but also in the presence of underlying pathological conditions (maternal PE).

On the other hand, the personal history of hereditary or acquired TTP can arouse the suspicion of TTP, measurement of ADAMTS13 should also be performed, and in case of levels < 20%, TTP is suspected, and anti- ADAMTS13 autoantibodies should also be measured [48].

Neurological manifestations are common in both TTP and PE. In the case of seizures, it is essential to immediately perform a CT angiography and basic laboratory tests to exclude any other possible emergency causes, which put a pregnant woman at risk and require a different clinical approach, such as brain hemorrhage, hypoglycemia, and electrolyte imbalance. It is also essential to estimate the fetus’s condition and measure the fetal heart rate with the use of ultrasonography [42].

Further laboratory investigation is also required for the differential diagnosis (blood count, renal and liver function tests, LDH, total and indirect bilirubin). A microscopical blood smear investigation could indicate the presence of schistocytes, burr cells, or echinocytes and speaks for the diagnosis of microangiopathic hemolytic anemia (TTP, Diffuse Intravascular Coagulation or HELLP syndrome) [40,42].

#### 3.3.2. Management

First, action is needed to prevent seizures and achieve lower levels of arterial blood pressure, using medication that can safely be administered during pregnancy, such as diazepam, MgSO4, labetalol, and methyldopa [42].

Delivery is the treatment of choice in patients with HELLP syndrome or preeclampsia, and amelioration is expected 1–2 days postpartum. In case there is no resolution or even worse, there is a deterioration of the clinical manifestations or the laboratory parameters (persistent anemia, neurological symptoms, further thrombocytopenia) in the first days postpartum, TTP is the most probable diagnosis and plasmapheresis should be performed without any further delay. In the literature, there are several case reports in which TTP was diagnosed postpartum because of deteriorating clinical and laboratory parameters after delivery [42,43,44,45,46,47,48].

Whenever TTP diagnosis is not conclusive, trial-plasmapheresis is a therapeutical option that would be applied a couple of days before delivery, while the effect of corticosteroids and MgSO4 is expected to result in an increase in platelets count. Moreover, in the case of improvement, TTP is the most probable diagnosis, over HELLP syndrome or preeclampsia [46]. In fact, TTP is more likely to cause the pregnant woman’s death (10–20% of all cases) compared with HELLP syndrome [42].

It should be noted that an immediate initiation of treatment is necessary, even in case there are no results of ADAMTS13 activity, ADAMTS13 inhibitor, or autoantibody measurement. Those results can be evaluated at a second time and are likely to contribute to the confirmation of the diagnosis or the differential diagnosis between the hereditary and the acquired form of the disease [42]. Identifying the specific form of the disease can help us decide which is the optimal therapeutic option in each case—corticosteroids or rituximab in the acquired form and plasma administration or plasmapheresis in the hereditary [47]. Corticosteroids should also be combined with plasmapheresis in the beginning, until the acquired form of the disease is excluded with the negativity of anti-ADAMTS13 autoantibodies [45].

According to the protocol presented in the special report of Fakhouri et al., in the case of a pregnant woman or a woman who had given birth recently with signs of TMA, symptomatic treatment and delivery are the best options, if typical findings of preeclampsia or HELLP syndrome are present. If the diagnosis is not precise and there are no neurological manifestations or signs of cardiac involvement, the woman should be closely monitored for 24–72 h. If there is no resolution of the clinical manifestations or improvement of the laboratory tests, or in case of neurological or heart involvement from the beginning, plasmapheresis should be initiated immediately with the administration of fresh frozen plasma [49].

## 4. Materials and Methods

We performed computer-assisted advanced research in the National Library of Medicine “Pubmed” and “Scopus” databases with the use of the following keywords: “preeclampsia/HELLP syndrome and ADAMTS-13”, “preeclampsia/HELLP syndrome and von Willebrand”, and “preeclampsia/HELLP syndrome and complement”. The only inclusion criteria applied were English language and publication date after the year 2005, while no limitations were applied concerning the papers’ type. All the authors of the present paper continued with a focused evaluation of the obtained papers and in the case of lack of evidence, all the members of our team approved a consensus-based decision-making process as the most appropriate one.

## Figures and Tables

**Table 1:** Bibliographic data concerning the relationship between vWF/ADAMTS13 in preeclampsia/HELLP syndrome (PE = preeclampsia, HP = Healthy pregnancy, NP = non-pregnancy).

Authors (Publication Date)	Sample	*n*	Control Group	*n*	vWF	ADAMTS13
Kalem et al. (2017) [13]	PE	12	HP	24		Decrease
Chen et al. (2021) [24]	PE	54	HP NP	33	Hyperadhesive	Decrease Decrease
Stepanian et al. (2011) [14]	PE	140	HP	140	No difference	Decrease
Alpoim et al. (2011) [15]	PE	55	HP NP	35 50	Increase Increase	Decrease Decrease
Sabau et al. (2016) [16]	HELLP	16	-			<30% in 13%
Molvarec et al. (2009) [19]	PE	67	HP NP	70 59	Normal multimeric pattern of vWF Increased vWF:Ag	No difference
Yoshida et al. (2017) [20]	PE HELLP	5 8	HP	128	Increased vWF:Ag	No difference
Aref et al. (2013) [2]	PE	110	HP NP	50 10	Increase	Decrease
Xiao et al. (2017) [17]	PE		-			Decrease
Hulstein et al. (2006) [18]	HELLP	14	HP PE	9 6	Increase Increase	Decrease No difference
von Krogh et al. (2015) [21]	PE	500	HP	500		No difference (levels, genotype, allele, haplotype, levels)
Drury Stewart et al. (2014) [23]	NP	46	-		vWF: Ag increase vWFpp increase (later stage) No high molecular weight vWF multimers	
Molvarec et al. (2009) [22]	PE	93	HP	127	Increase	

**Table 2:** Mississippi and Tennessee systems for the classification of HELLP syndrome [42].

Mississippi System		Tennessee System
Class 1	PLTs < 50.000/mm^3^	Complete syndrome
Class 2	PLTs: 50.000–100.000/mm^3^	Incomplete syndrome
Class 3	-PLTs> 100.000–150.000/mm^3^ -Hemolysis and elevated liver enzymes—LDH > 600 IU/I	

**Table 3:** Comparison between HELLP syndrome and TTP in terms of various clinical and laboratory parameters, +: mild, ++: medium, +++: severe [41].

Parameters	HELLP Syndrome	TTP
Hemolysis	+ to +++	+++
Schistocytosis	+ to +++	+++
LDH	++ to +++	+++
Liver enzymes	++ to +++	No or +
Platelets	++ to +++	+++
Total Bilirubin	+	+ to ++
Proteinuria	+++	+ to ++
ADAMTS13	Detectable (decreased)	Undetectable
Fever	No	++

**Table 4:** Collected bibliographic data concerning published case reports with complicated differential diagnosis [43,44,45,46,47,48].

Authors Age (Years)/Gestational Week	Signs/Symptoms of Admission	Initial Diagnosis	Clinical Course	Final Diagnosis
Ramadan et al. (2018) 26 y/33 w [43]	Seizures high blood pressure	HELLP	PLTs decrease and LDH increase on postpartum day 6 after initial amelioration	HELLP & TTP
Ramadan et al. (2018) 36 y/27 w [43]	Thrombocytopenia	Acquired TTP	headache, epigastric pain, and tachypnea manifestation, high blood pressure	TTP& PE/HELLP
Ramadan et al. (2018) 24 y/37 w [43]	High blood pressure, edema, mild anemia	PE	hypertension, headache, thrombocytopenia, hyperuricemia, SGOT, serum creatinine, and LDH elevation, schistocytes	PE & TTP
González-Mesa et al. (2013) 30 y/28 w [48]	Dizziness, headache, Other neurological manifestation, anemia, thrombocytopenia	TTP	Dyspnea, hypoxia, cardiopulmonary arrest	TTP
Ehsanipoor et al. (2005) 26 y/22 w [44]	epigastric pain, proteinuria, and elevated blood pressure	HELLP	persistent thrombocytopenia and hemolytic anemia	HELLP & TTP
Patrick et al. (2012) 28 y/32.2 w [45]	Headache Elevated blood pressure	TTP	Persistent thrombocytopenia, liver enzymes elevation	TTP& PE
Mousseaux et al. (2020) 28 y/37 w [46]	Elevated Blood pressure, proteinuria	PE	Epigastric pain, fetal bradycardia, anuria (after delivery)	PE &TTP
Bhute et al. (2020) 25 y/34 w [47]	Headache, seizures, jaundice, high blood pressure, edema	HELLP	Worsening proteinuria, liver enzymes elevation	HELLP & TTP

## Data Availability

Not applicable.
